# Supplementary material for: Impact of Evacuation on the Long-Term Trend of Metabolic Syndrome after the Great East Japan Earthquake
Source: Int J Environ Res Public Health. 2022 Aug 2;19(15):9492. doi: 10.3390/ijerph19159492 (PMC9368087; doi:10.3390/ijerph19159492)
Supplement: Supplementary file 1 [file ijerph-19-09492-s001.zip › ijerph-1781000-supplementary.pdf]

International Journal of
Environmental Research and Public Health

MDPI

Supplemental tables

Table S1. Prevalence of metabolic syndrome (international criteria) according to fiscal years and areas stratified by gender and age category.

	2008	2009	2010	2011	2012	2013	2014	2015	2016	2017
**Total**										
**Mountainous area**	26.5	26.1	26.3	26.8	27.2	27.4	27.9	27.8	28.8	28.6
**Central area**	24.4	24.1	24.1	24.5	25.0	24.8	25.3	25.4	26.1	26.5
**Coastal area**	25.9	25.0	25.5	25.8	26.6	26.6	26.6	26.5	27.3	27.7
**Evacuation area**	26.8	26.1	26.7	29.0	31.1	31.7	31.9	32.2	32.9	32.6
**Men**										
**Mountainous area**	25.6	25.9	26.0	27.2	27.6	28.0	28.8	28.7	30.1	30.0
**Central area**	23.9	24.0	24.2	24.9	25.6	25.5	26.2	26.4	27.1	28.0
**Coastal area**	26.3	25.5	26.4	27.2	28.0	28.1	28.2	28.1	29.1	29.9
**Evacuation area**	24.7	24.6	24.9	28.0	30.4	31.2	31.6	32.3	33.2	33.3
**Women**										
**Mountainous area**	27.2	26.1	26.4	26.3	26.6	26.8	27.1	26.9	27.5	27.4
**Central area**	24.9	24.0	23.9	23.9	24.3	24.0	24.2	24.3	25.0	24.9
**Coastal area**	25.2	24.1	24.4	23.9	25.0	24.8	24.8	24.7	25.3	25.3
**Evacuation area**	28.9	27.6	28.5	29.9	31.9	32.1	32.2	32.3	32.5	31.7
**Age <60 years**										
**Mountainous area**	21.0	20.6	20.5	20.9	21.2	21.2	21.8	21.4	21.9	22.3
**Central area**	19.4	19.2	19.3	19.3	19.7	19.4	19.8	19.7	20.3	20.5
**Coastal area**	21.6	20.5	20.9	21.1	21.9	21.8	21.6	21.1	21.8	22.2
**Evacuation area**	21.3	20.6	20.7	22.6	24.7	25.1	25.1	25.0	25.5	25.6
**Age ≥60 years**										
**Mountainous area**	34.5	34.1	34.4	34.8	35.3	35.8	36.3	36.4	37.8	37.1
**Central area**	33.4	32.7	32.6	33.1	33.9	34.1	34.6	34.9	35.9	36.5
**Coastal area**	34.1	33.0	33.7	33.8	34.7	34.8	35.3	35.8	36.6	37.1
**Evacuation area**	33.9	33.1	34.2	36.7	39.1	39.8	40.2	41.1	41.7	41.0

Table S2. Risk ratios and 95% confidence intervals of metabolic syndrome after the Great East Japan Earthquake compared to that before the disaster (international criteria).

	2008–2010	2011	2012	2013	2014	2015	2016	2017
**Total**								
**Mountainous area**	1.00	1.02 (1.00–1.04)	1.03 (1.02–1.05)	1.04 (1.02–1.06)	1.06 (1.04–1.08)	1.06 (1.04–1.08)	1.10 (1.08–1.12)	1.09 (1.07–1.11)
**Central area**	1.00	1.01 (1.00–1.02)	1.03 (1.02–1.04)	1.03 (1.02–1.04)	1.04 (1.03–1.05)	1.05 (1.04–1.06)	1.08 (1.07–1.09)	1.09 (1.08–1.10)
**Coastal area**	1.00	1.01 (0.99–1.03)	1.05 (1.03–1.06)	1.04 (1.03–1.06)	1.04 (1.03–1.06)	1.04 (1.02–1.05)	1.07 (1.05–1.09)	1.08 (1.07–1.10)
**Evacuation area**	1.00	1.10 (1.07–1.13)	1.18 (1.15–1.21)	1.20 (1.17–1.23)	1.20 (1.18–1.23)	1.22 (1.19–1.24)	1.24 (1.22–1.27)	1.23 (1.20–1.25)
**Men**								
**Mountainous area**	1.00	1.05 (1.03–1.08)	1.07 (1.04–1.10)	1.08 (1.05–1.11)	1.11 (1.09–1.14)	1.11 (1.08–1.14)	1.16 (1.14–1.19)	1.16 (1.13–1.19)
**Central area**	1.00	1.03 (1.02–1.05)	1.06 (1.05–1.08)	1.06 (1.04–1.07)	1.08 (1.07–1.10)	1.09 (1.08–1.10)	1.12 (1.11–1.14)	1.16 (1.14–1.17)
**Coastal area**	1.00	1.04 (1.02–1.07)	1.07 (1.05–1.10)	1.07 (1.05–1.10)	1.08 (1.05–1.10)	1.07 (1.05–1.09)	1.11 (1.09–1.13)	1.14 (1.11–1.16)
**Evacuation area**	1.00	1.13 (1.09–1.17)	1.23 (1.19–1.27)	1.26 (1.22–1.30)	1.27 (1.23–1.31)	1.30 (1.26–1.34)	1.33 (1.29–1.37)	1.33 (1.30–1.38)
**Women**								
**Mountainous area**	1.00	0.99 (0.97–1.02)	1.01 (0.98–1.03)	1.01 (0.99–1.04)	1.02 (1.00–1.05)	1.02 (0.99–1.04)	1.04 (1.02–1.07)	1.04 (1.01–1.07)
**Central area**	1.00	0.99 (0.98–1.01)	1.01 (0.99–1.02)	0.99 (0.98–1.01)	1.00 (0.99–1.02)	1.01 (0.99–1.02)	1.04 (1.02–1.05)	1.03 (1.02–1.04)
**Coastal area**	1.00	0.97 (0.95–1.00)	1.02 (0.99–1.05)	1.01 (0.99–1.04)	1.01 (0.98–1.03)	1.00 (0.98–1.03)	1.03 (1.01–1.06)	1.03 (1.00–1.06)
**Evacuation area**	1.00	1.06 (1.02–1.10)	1.13 (1.10–1.17)	1.14 (1.11–1.18)	1.14 (1.11–1.18)	1.15 (1.11–1.18)	1.16 (1.12–1.19)	1.13 (1.09–1.16)
**Age <60 years**								
**Mountainous area**	1.00	1.01 (0.99–1.04)	1.02 (1.00–1.05)	1.03 (1.00–1.05)	1.06 (1.03–1.08)	1.03 (1.01–1.06)	1.06 (1.03–1.09)	1.08 (1.05–1.11)
**Central area**	1.00	1.00 (0.99–1.02)	1.02 (1.01–1.03)	1.01 (0.99–1.02)	1.03 (1.01–1.04)	1.02 (1.01–1.04)	1.05 (1.04–1.06)	1.07 (1.05–1.08)
**Coastal area**	1.00	1.00 (0.98–1.03)	1.04 (1.02–1.07)	1.04 (1.01–1.06)	1.03 (1.00–1.05)	1.00 (0.98–1.03)	1.04 (1.02–1.06)	1.06 (1.04–1.08)
**Evacuation area**	1.00	1.08 (1.04–1.12)	1.18 (1.14–1.22)	1.20 (1.16–1.24)	1.21 (1.17–1.25)	1.20 (1.16–1.24)	1.22 (1.19–1.27)	1.23 (1.19–1.27)
**Age ≥60 years**								
**Mountainous area**	1.00	1.01 (0.99–1.04)	1.03 (1.00–1.06)	1.04 (1.02–1.07)	1.06 (1.03–1.08)	1.06 (1.04–1.09)	1.10 (1.08–1.13)	1.08 (1.06–1.11)
**Central area**	1.00	1.01 (0.99–1.02)	1.03 (1.02–1.05)	1.04 (1.02–1.05)	1.05 (1.04–1.07)	1.06 (1.05–1.08)	1.09 (1.08–1.11)	1.11 (1.09–1.12)
**Coastal area**	1.00	1.01 (0.98–1.03)	1.03 (1.01–1.06)	1.04 (1.01–1.06)	1.05 (1.03–1.08)	1.06 (1.04–1.09)	1.09 (1.06–1.11)	1.10 (1.08–1.13)
**Evacuation area**	1.00	1.09 (1.05–1.13)	1.16 (1.12–1.20)	1.18 (1.14–1.21)	1.19 (1.16–1.23)	1.22 (1.18–1.25)	1.24 (1.20–1.27)	1.21 (1.18–1.25)

Table S3. Risk ratios and 95% confidence intervals of metabolic syndrome (international criteria) for the evacuation area compared with the mountainous area in 2008–2017.

	2008	2009	2010	2011	2012
**Total**					
**Mountainous area**	1.00	1.00	1.00	1.00	1.00
**Central area**	0.96 (0.94–0.98)	0.96 (0.94–0.98)	0.95 (0.94–0.97)	0.95 (0.93–0.97)	0.95 (0.94–0.97)
**Coastal area**	1.02 (1.00–1.04)	1.00 (0.98–1.02)	1.01 (0.99–1.04)	1.00 (0.98–1.02)	1.02 (1.00–1.04)

**Evacuation area**	1.00 (0.97–1.02)	0.99 (0.96–1.01)	1.00 (0.98–1.03)	1.07 (1.04–1.10)	1.13 (1.10–1.16)
**Men**					
**Mountainous area**	1.00	1.00	1.00	1.00	1.00
**Central area**	0.95 (0.92–0.98)	0.94 (0.92–0.97)	0.95 (0.93–0.98)	0.93 (0.91–0.96)	0.94 (0.92–0.97)
**Coastal area**	1.05 (1.02–1.09)	1.01 (0.97–1.04)	1.04 (1.01–1.07)	1.03 (1.00–1.06)	1.04 (1.01–1.07)
**Evacuation area**	0.96 (0.92–1.00)	0.94 (0.91–0.98)	0.95 (0.92–0.99)	1.02 (0.99–1.06)	1.09 (1.05–1.13)
**Women**					
**Mountainous area**	1.00	1.00	1.00	1.00	1.00
**Central area**	0.97 (0.95–1.00)	0.97 (0.95–1.00)	0.96 (0.94–0.99)	0.96 (0.94–0.99)	0.97 (0.94–0.99)
**Coastal area**	0.98 (0.95–1.01)	0.97 (0.94–1.01)	0.97 (0.94–1.00)	0.96 (0.92–0.99)	0.98 (0.95–1.02)
**Evacuation area**	1.05 (1.01–1.09)	1.05 (1.01–1.08)	1.07 (1.03–1.11)	1.13 (1.08–1.17)	1.18 (1.14–1.23)
**Age <60 years**					
**Mountainous area**	1.00	1.00	1.00	1.00	1.00
**Central area**	0.92 (0.89–0.94)	0.92 (0.90–0.95)	0.93 (0.90–0.96)	0.91 (0.89–0.94)	0.92 (0.89–0.94)
**Coastal area**	1.01 (0.98–1.05)	0.98 (0.95–1.01)	1.00 (0.97–1.04)	0.99 (0.96–1.02)	1.01 (0.98–1.04)
**Evacuation area**	1.01 (0.97–1.05)	1.00 (0.96–1.04)	1.01 (0.97–1.05)	1.07 (1.03–1.11)	1.16 (1.11–1.20)
**Age ≥60 years**					
**Mountainous area**	1.00	1.00	1.00	1.00	1.00
**Central area**	0.98 (0.95–1.00)	0.97 (0.94–0.99)	0.95 (0.93–0.98)	0.96 (0.93–0.98)	0.96 (0.94–0.99)
**Coastal area**	1.00 (0.96–1.03)	0.97 (0.94–1.01)	0.99 (0.96–1.02)	0.98 (0.95–1.01)	0.99 (0.96–1.02)
**Evacuation area**	0.99 (0.96–1.03)	0.98 (0.95–1.02)	1.00 (0.97–1.04)	1.06 (1.02–1.10)	1.11 (1.08–1.15)

	2013	2014	2015	2016	2017
**Total**					
**Mountainous area**	1.00	1.00	1.00	1.00	1.00
**Central area**	0.94 (0.93–0.96)	0.94 (0.92–0.95)	0.95 (0.93–0.96)	0.94 (0.93–0.96)	0.96 (0.94–0.97)
**Coastal area**	1.01 (0.99–1.03)	0.99 (0.97–1.01)	0.99 (0.97–1.01)	0.98 (0.97–1.00)	1.00 (0.98–1.02)
**Evacuation area**	1.14 (1.11–1.17)	1.12 (1.10–1.15)	1.14 (1.11–1.17)	1.12 (1.10–1.15)	1.11 (1.09–1.14)
**Men**					
**Mountainous area**	1.00	1.00	1.00	1.00	1.00
**Central area**	0.93 (0.91–0.96)	0.93 (0.91–0.95)	0.94 (0.92–0.96)	0.92 (0.90–0.94)	0.96 (0.94–0.98)
**Coastal area**	1.03 (1.00–1.06)	1.01 (0.98–1.03)	1.01 (0.98–1.03)	0.99 (0.97–1.02)	1.03 (1.00–1.05)
**Evacuation area**	1.10 (1.07–1.14)	1.08 (1.05–1.12)	1.11 (1.07–1.14)	1.09 (1.05–1.12)	1.09 (1.06–1.13)
**Women**					
**Mountainous area**	1.00	1.00	1.00	1.00	1.00
**Central area**	0.95 (0.93–0.97)	0.94 (0.92–0.97)	0.96 (0.93–0.98)	0.96 (0.94–0.98)	0.96 (0.93–0.98)
**Coastal area**	0.97 (0.94–1.00)	0.96 (0.93–0.99)	0.96 (0.93–0.99)	0.96 (0.93–0.99)	0.96 (0.93–0.99)
**Evacuation area**	1.19 (1.14–1.23)	1.17 (1.13–1.21)	1.18 (1.14–1.22)	1.16 (1.12–1.20)	1.14 (1.10–1.18)
**Age <60 years**					
**Mountainous area**	1.00	1.00	1.00	1.00	1.00
**Central area**	0.90 (0.88–0.93)	0.90 (0.87–0.92)	0.91 (0.89–0.93)	0.91 (0.89–0.93)	0.91 (0.89–0.93)
**Coastal area**	1.01 (0.97–1.04)	0.97 (0.94–1.00)	0.96 (0.94–0.99)	0.97 (0.94–1.00)	0.98 (0.95–1.00)
**Evacuation area**	1.17 (1.13–1.22)	1.15 (1.11–1.19)	1.16 (1.12–1.20)	1.16 (1.12–1.20)	1.14 (1.10–1.18)
**Age ≥60 years**					
**Mountainous area**	1.00	1.00	1.00	1.00	1.00
**Central area**	0.95 (0.93–0.98)	0.96 (0.93–0.98)	0.96 (0.94–0.99)	0.95 (0.93–0.97)	0.99 (0.96–1.01)
**Coastal area**	0.98 (0.95–1.00)	0.98 (0.95–1.01)	0.99 (0.96–1.01)	0.97 (0.95–1.00)	1.00 (0.98–1.03)
**Evacuation area**	1.11 (1.08–1.15)	1.11 (1.08–1.15)	1.13 (1.10–1.17)	1.11 (1.07–1.14)	1.11 (1.08–1.14)
